# Use of Oral Empagliflozin to Obtain Optimal Blood Sugar Levels for Conducting ^18^ F-FDG PET-CT in Patients with Hyperglycemia—A Pilot Study

**DOI:** 10.1055/s-0043-1771283

**Published:** 2023-09-06

**Authors:** Abhishek Mahato, Anurag Jain, V.S Prakash, Rajesh Nair, Richa Joshi, Dharmesh Paliwal, Awadhesh Tiwari, Sukhanshi Khandpur, Harkirat Singh

**Affiliations:** 1 Department of Nuclear Medicine, Command Hospital, Lucknow, Uttar Pradesh, India; 2 Department of Medical Oncology, Command Hospital, Lucknow, Uttar Pradesh, India; 3 Department of Surgical Oncology, Command Hospital, Lucknow, Uttar Pradesh, India; 4 Department of Nuclear Medicine, Command Hospital, Pune, Maharashtra, India; 5 Department of Biostatistics, Sanjay Gandhi Post Graduate Institute of Medical Sciences (SGPGI), Lucknow, Uttar Pradesh, India

**Keywords:** ^18^ F-FDG, empagliflozin, optimal blood sugar, PET-CT, SGLT2

## Abstract

**Background** Flourine-18 fluorodeoxyglucose positron emission tomography-computed tomography ( ^18^ F-FDG PET-CT) is a well-established imaging modality for the evaluation of patients with oncological and nononcological conditions. The underlying principle of imaging is the preferentially increased glucose consumption by cancer cells, due to overexpression of glucose type 1 receptors that are insulin independent. Thus, one of the factors that leads to decreased sensitivity of an ^18^ F-FDG PET-CT is elevated blood sugar levels, leading to decreased glucose uptake by cancer cells due to competitive inhibition.

A significant percentage of patients scheduled for PET-CT scan has diabetes mellitus type II as a comorbid condition and often has elevated random blood sugar (RBS) precluding an upfront PET-CT evaluation. Such cases must be rescheduled. This causes delay in the evaluation and management of such patients.

Empagliflozin is a novel sodium glucose type 2 inhibitor that prevents tubular reabsorption of glucose and increases renal glycosuria resulting in decreased blood sugar. This drug does not cause significant hypoglycemia or increase endogenous insulin secretion. This study was undertaken to evaluate a potential role for empagliflozin in facilitating optimal blood sugar control in patients with hyperglycemia on the day of the scheduled PET scan.

**Methods** This is an interventional prospective study and patients detected to have RBS more than 200 mg/dL on the day of the scheduled scan were included in the study. The patients were administered two tablets of 10 mg empagliflozin and kept under observation. Samples for RBS were taken at approximately 2nd and 4th hour post administration by bedside method. These patients underwent scan on the same day after adequate sugar control and when an RBS of less than 200 mg/dL was achieved. The primary outcome studied was change in RBS values in the patient cohort and evaluation of PET SUV (standardized uptake value) compared with the rest of the patients scheduled on the same day. Secondary outcome was assessment of any side effects in the patients.

**Results** Total of 10 patients were found to have elevated blood sugar (RBS > 200 mg/dL; irrespective of being on medication) and did not meet the evaluation criteria for a PET-CT scan on the scheduled day. Following administration of the drug, all 10 patients were able to attain blood sugar levels and fulfill the criteria for undergoing a PET-CT scan. No obvious side effect was noted in any of the patient. The SUV values of the patient cohort were comparable with the rest of the patient scanned on the day.

**Conclusion** In this pilot study, 20 mg of empagliflozin (2 tablets of 10 mg) appears to be a safe and effective method for achieving optimal decrease in the RBS without causing hypoglycemia or hyperinsulinemia. It can be safely employed in the subset of population with RBS between 201 and 300 mg/dL to adequately bring the sugar levels at acceptable levels RBS less than 200 mg/dl and fulfill the FDG PET-CT criteria as per European Association of Nuclear Medicine (EANM) norms.

## Introduction

Flourine-18 fluorodeoxyglucose positron emission tomography-computed tomography ( ^18^ F-FDG PET-CT) is a well-established modality providing tomographic images for the assessment of metabolic activity in the tumor and inflammatory tissues. ^18^ F-FDG is a glucose isomer with a half-life of 109.7 minutes. This radio-tracer undergoes metabolic trapping in the cells and achieves higher concentration in the tumor and inflammatory cells. This is due to overexpression of glucose type 1 receptors and increased hexokinase activity in these cells. ^18^ F-FDG undergoes competitive inhibition by increased blood glucose and redistribution to skeletal muscles because of insulin. Hence, hyperglycemia and hyperinsulinemia decrease the sensitivity of the FDG PET-CT scan due to decreased uptake of ^18^ F-FDG in tumor cells and increase of background activity respectively.

EANM procedure guidelines 2.0 for FDG PET-CT suggests at least 4 hours of fasting in nondiabetics to ensure low blood glucose and insulin levels. 1 It recommends measurement of random blood sugar (RBS) before FDG administration using a glucometer. 2 The ideal RBS as per the guidelines should be between 4 and 7 mmol/L and 70 to 200 mg/dL for a PET-CT scan. As it has been shown in a meta-analysis that the standardized uptake values (SUVs) of brain and muscles were significantly higher and SUVs of the liver and blood pool were significantly lower in the hyperglycemic group as compared to euglycemic patients. 3 Hence, for values of FDG more than 200 mg/dL guidelines recommend rescheduling or excluding the patient from a clinical study.

Some studies have used subcutaneous injections of rapid insulin to control the blood sugar but that causes delay in FDG administration by up to 4 hours followed by a late morning scan. Studies have also used intravenous insulin; however, this has not yet been validated. 4 Moreover, use of insulin has the risk of causing hypoglycemia and skeletal muscle uptake. The guidelines recommend discontinuing metformin for 48 hours prior if patient is scheduled for a scan with intravenous contrast agent. This scenario would also present with fasting hyperglycemia; the guidelines recommend use of an alternative for blood sugar control. 5 6 7

Since PET-CT is required for detection, staging, restaging, and therapy response assessment, rescheduling or exclusion of a PET-CT study causes delay in the management of patients. The impact of this delay is more in developing countries where access to healthcare is still very limited. Our aim was to study the potential use of sodium glucose type 2 (SGLT2) inhibitor empagliflozin for reducing blood sugar levels in such a patient subset.

Empagliflozin is a SGLT2 inhibitor approved by U.S. Food and Drug Administration (FDA) for use in type II diabetes mellitus (T2DM) with preserved renal function. Empagliflozin is an oral formulation available in 10 and 25 mg tablets for once daily use. It decreases the renal tubular reabsorption by lowering renal threshold and increasing urinary excretion of glucose. The onset of action of empagliflozin is approximately 1.5 hours (90 minutes) and elimination half-life is 12.4 hours. 8 The drug can be safely administered in patients with normal—mildly impaired—glomerular filtration rate and does not cause hypoglycemic symptoms in isolation.

## Materials and Methods

The study was performed in the Department of Nuclear Medicine at Command Hospital Lucknow and Pune. No funding of any kind was utilized for conducting this study. The study protocol was approved by the institutional ethical committee. The study was conducted in accordance with the principles of the Declaration of Helsinki. The authors assume responsibility for the accuracy and completeness of the data and analyses, as well as for the fidelity of the study and this report to the protocol.

The study was performed between January 2022 and April 2023. The inclusion criteria were (1) hyperglycemia with RBS more than 200 mg/dL, (2) normal serum creatinine values, and (3) Karnofsky performance score less than 2.

A total of 10 patients met the inclusion criteria in the study period. After double confirmation of the raised RBS at baseline, the patients were administered two 10 mg tablets of empagliflozin. RBS was measured at an interval of approximately 2 hours at 2nd and 4th hour after drug administration. Maximum time limit of 4 hours was taken as cutoff for the study taking into consideration the half-life of ^18^ F-FDG and department functionality.

Patients were under constant vigilance and were asked to report any symptoms of hypoglycemia. The patients were administered FDG if the RBS levels were below 200 mg/dL.

## Results

Total of 10 patients met the criteria for inclusion in the study ( Table 1 ). The patients were eight females and two males with mean age of 58.3 (46–69) years. One patient was a newly detected diabetes during the routine test, while the rest of the patients were known diabetics on medication. All patients had normal renal function with mean serum creatinine values of 0.67 (0.3–1.0) mg/dL.

**Table 1:** Presenting patient attributes alongside the temporal evolution of blood sugar following the administration of Empagliflozin tablets

Patient	Date	Age	Gender	S. Cr	RBS baseline	RBS 2	RBS 3	Time baseline	Time 2	Time 3	Delta RBS	Delta time in minutes
P1	24/02/22	64	F	0.67	209	159	0	9:30	12:15	—	50	165
P2	15/02/22	46	F	0.5	248	168	0	9:20	10:55	—	80	95
P3	15/02/22	51	F	0.3	236	91	0	13:00	14:20	—	145	80
P4	25/02/22	52	F	0.8	207	201	180	10:10	11:15	12:28	27	138
P5	02/06/22	57	F	0.6	296	212	158	8:38	10:38	12:00	138	120
P6	24/01/23	47	F	0.8	214	165	0	9:18	11:18	—	49	120
P7	22/2/23	68	M	1.0	218	157	0	9:00	11:00	—	61	120
P8	20/2/23	64	F	0.7	255	210	190	8:45	11:00	12:08	65	213
P9	8/2/23	69	F	0.6	207	136	0	8:55	11:25	—	71	150
P10	10/4/23	65	M	0.7	219	152	0	9:25	11:55	—	67	150
Average	—	58.3	—	0.667	230.9	—	—	—	—	Average	75.3	135.1

Abbreviation: RBS, random blood sugar.

The mean RBS of the patient cohort was 230.9 (207–296) mg/dL. The average reduction in RBS was 75.3 (27–145) mg/dL. The average time for achieving the target RBS of less than 200 mg/dL was achieved in a mean time interval of 135.1 (80–213) minutes. No significant difference was noted in the SUV values of the brain parenchyma ( *p* = 0.49) and blood pool activity ( *p* = 0.41) when compared with the nonmedicated females of the scheduled day. SUV values of the liver were higher in the medicated arm with significant difference between the medicated arm ( *p* < 0.01; Table 2 ). All the primary lesion and metastatic sites could be identified in the patient cohort. The patient did not report any side effects during the day of test and within a duration of 1 week post administration.

**Table 2:** Statistical comparison of SUV values of medicated and non-medicated patients

SUV	Median (IQR)		*p* -Value
	Medicated patients	Nonmedicated patients	
Blood pool	2.30 (2.10–2.40)	2.14 (1.88–2.35)	0.41
Brain	9.50 (8.25–10.20)	10.35 (7.63–12.41)	0.49
Liver	3.50 (2.90–3.85)	2.61 (2.39–3.07)	0.01

Abbreviations: IQR, interquartile range; SUV, standardized uptake value.

Note: The average liver SUV of medicated patients is significantly higher than nonmedicated patients (median (IQR): 3.50 [2.90–3.85] vs. 2.61 [2.39–3.07], *p* = 0.01). No statistically significant difference was observed in SUV of liver and brain when evaluated using Mann–Whitney U test.

## Discussion

Empagliflozin along with canagliflozin and dapagliflozin is a new class of oral hypoglycemic drugs that act by SGLT2 inhibition. Glucose undergoes glomerular filtration and SGLT2 mediates 90% of the tubular reabsorption of glucose. 9 Inhibition of SGLT2 causes decrease in the tubular reabsorption of glucose resulting in increased renal excretion and decreased blood glucose. This action of SGLT2 inhibition does not affect the insulin pathway. Empagliflozin is approved by FDA for the treatment of T2DM in patient with normal renal function, absence of urinary tract infection, and normal body mass index. 10 It also has highest SGLT2 selectivity when compared to canagliflozin and dapagliflozin. 11 Empagliflozin acts by increasing renal excretion of glucose, improving the beta cell function, and increasing utilization of lipids over glucose. 8

In our study, we found that 20 mg empagliflozin was able to reduce the RBS values by 36% (13–61%) and mean reduction of 75.3 mg/dL (27–145 mg/dL) in the patient cohort with the mean RBS of 230.9 (207–296) mg/dL. This matches with the findings shown in other studies where a single dose of empagliflozin was shown to cause 36 to 45% decrease in glucose absorption with increase in glucose excretion by 11-fold in a 10 mg dose to 18-fold in 25 mg dose. 8 12 The drug is recommended to be used once daily in the morning with or without food; however, decrease to the tune of 37% is noted in the peak concentration of drug after a heavy meal when compared to the fasting state. 13

The onset of action of empagliflozin is around 90 minutes and this shows in our study wherein the average time for reduction was around 135.1 (80–213) minutes. This is better than the average waiting time of 4 hours before FDG administration after the use of rapid insulin for controlling hyperglycemia as per EANM guidelines.

None of the patient had any side effect following administration of 20 mg (two tablets of 10 mg) empagliflozin. No significant difference was noted in the SUV max values in the brain, liver, mediastinal blood pool of the medicated patients when compared with the SUV values of the other patient scanned on that day. There was no obvious increase in skeletal muscle uptake in any of the patient cohort. Insulin use, in comparison to tablet Empagliflozin, results in up to 4-hour FDG administration delay, induces skeletal muscle uptake, and poses a risk of hypoglycemia in patients.

## Conclusion

With the help of this study, we would like to emphasize on the fact that 20 mg of empagliflozin (2 tablets of 10 mg) can reduce the RBS values by 36% (13–61%). This can be successfully used to control hyperglycemia in patients with preserved renal function and deranged RBS values of up to 300 mg/dL on the day of the scheduled FDG PET-CT scan. Use of empagliflozin does not alter the sensitivity of FDG PET-CT scan in the patient subset and has no side effect when administered in a single dose. Due to the absence of any side effect, the drug can also be safely used in standalone diagnostic centers lacking emergency healthcare facility. The use of drug has a potential role in avoiding delay of patients scan and subsequent treatment. Moreover, the higher drug concentration when administered in a fasting state syncs with the fasting protocol of an FDG PET-CT scan. Similar study with larger patient subset can provide us with a more comprehensive answer on this potentially useful intervention.
